# Supplementary material for: PinkyCaMP: an mScarlet-based calcium sensor with enhanced brightness, photostability and multiplexing capabilities
Source: Nat Methods. 2026 Apr 24;23(5):998–1010. doi: 10.1038/s41592-026-03065-2 (PMC13167472; doi:10.1038/s41592-026-03065-2)
Supplement: Supplementary file 1 — Supplementary Figs. 1–5. [file 41592_2026_3065_MOESM1_ESM.pdf]

nature methods

Supplementary information https://doi.org/10.1038/s41592-026-03065-2

PinkyCaMP: an mScarlet-based calcium sensor with enhanced brightness, photostability and multiplexing capabilities

In the format provided by the authors and unedited

Supplementary Material

Table of Contents

Supplementary Figure 1: Sequence alignment of mScarlet, R-GECO1 and PinkyCaMP variants

Supplementary Figure 2: *In vitro* characterization of PinkyCaMP variants.

Supplementary Figure 3: pH dependence of PinkyCaMP variants.

Supplementary Figure 4: Characterization of PinkyCaMP and other GECIs in organotypic slice cultures.

Supplementary Figure 5: Comparison of PinkyCaMP to other red GECIs in brain slices.

Supplementary Figures

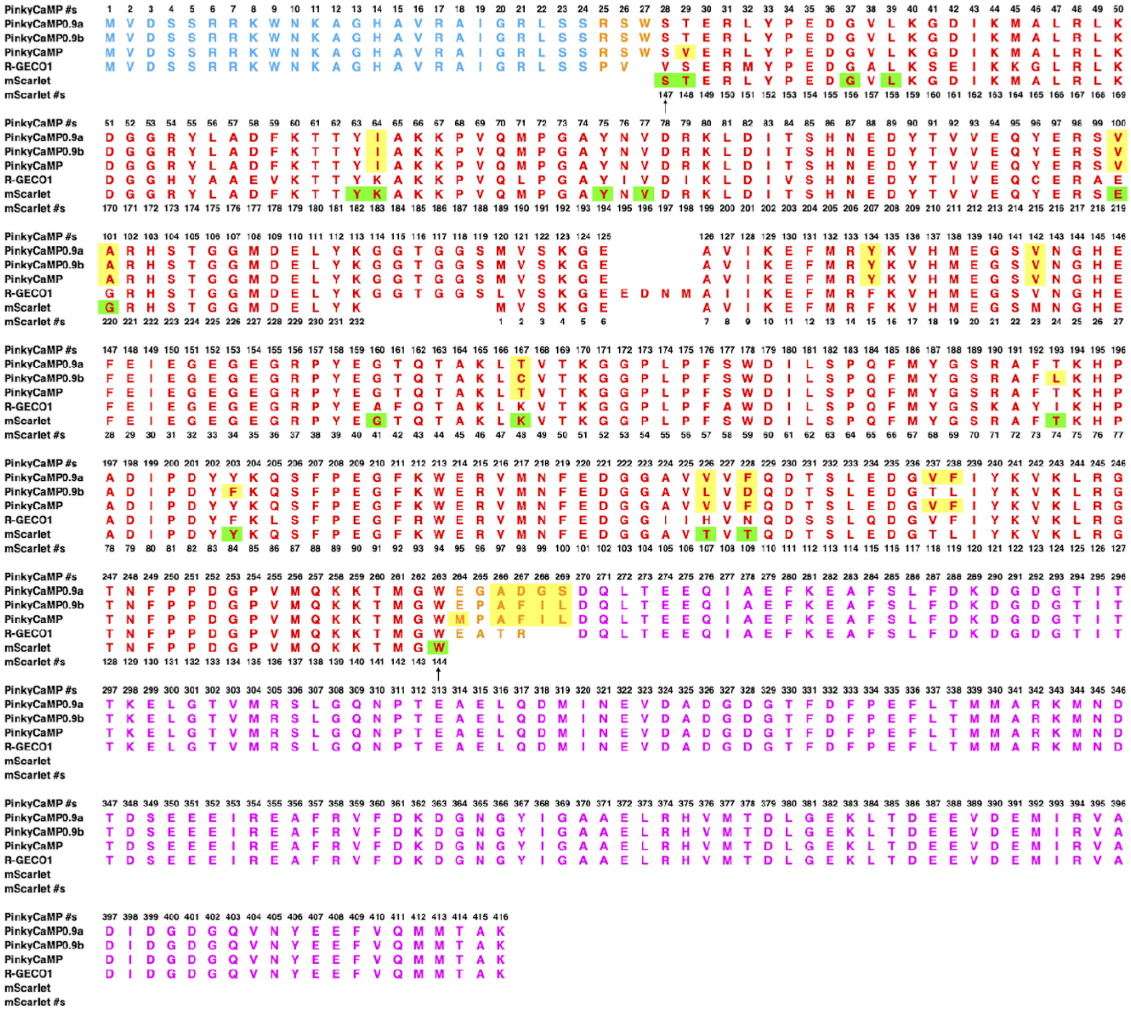

Supplementary Figure 1: Sequence alignment of mScarlet, R-GECO1 and PinkyCaMP variants. The mScarlet gene sequence has been permuted in order to align with the cpmScarlet domain of PinkyCaMP and the mApple domain of R-GECO1. RS20 is colored in cyan, the FP domain is colored in red, CaM is colored in magenta and the linkers are colored in orange. The mutations accumulated during the optimization process are highlighted in yellow. The residues of mScarlet subjected to site-saturation mutagenesis are highlighted in green. During site-saturation mutagenesis, the original residue was identified as the preferred residue at some positions. The gate post residues are indicated by arrows.

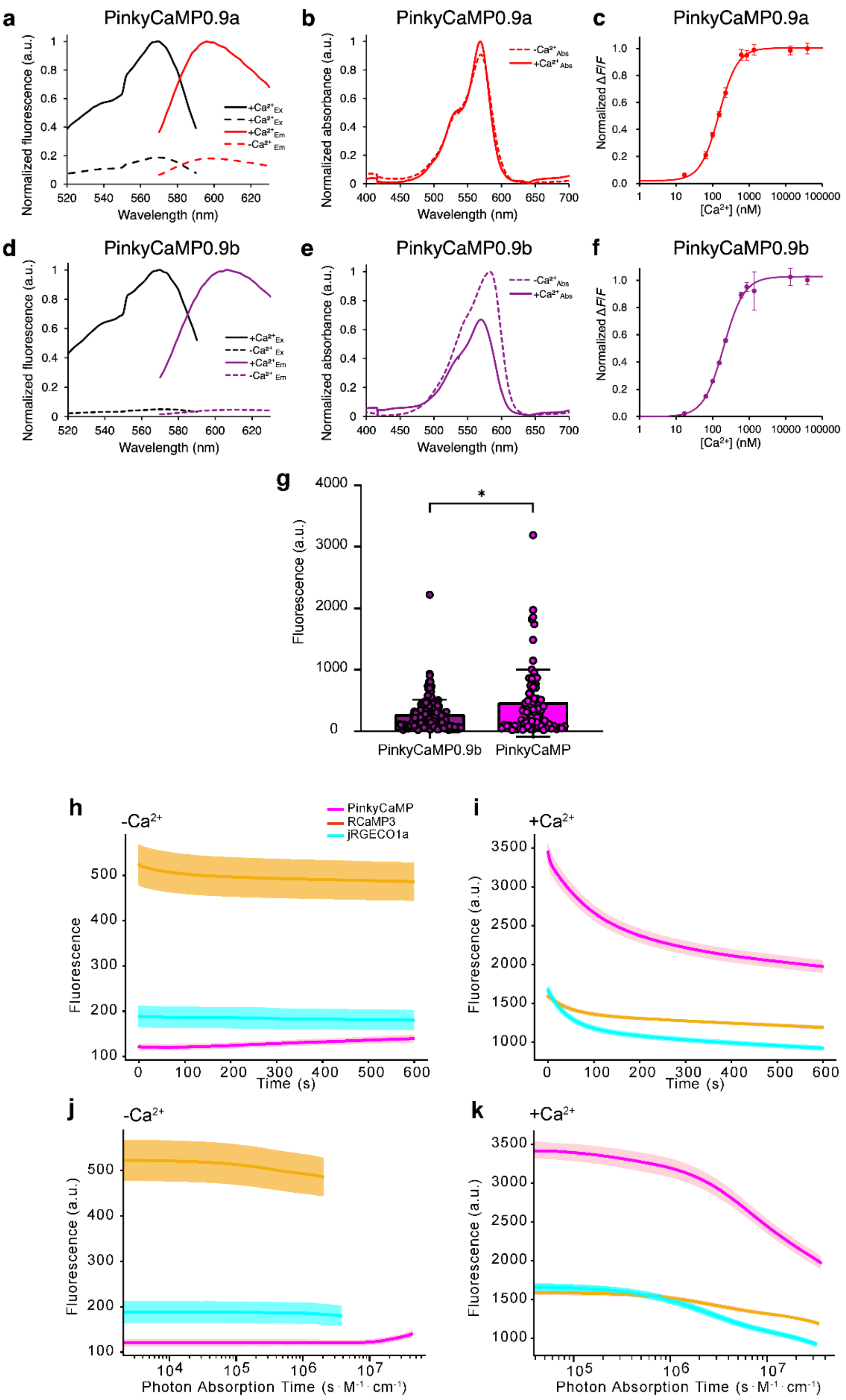

Supplementary Fig. 2: *In vitro* characterization of PinkyCaMP variants. The characterization of PinkyCaMP0.9c (= PinkyCaMP) is provided in **Figure 1d,e,f**. (**a**,**d**) Excitation (emission at 630 nm), and emission (excitation at 535 nm) spectra in the presence (39 µM) and absence of Ca^{2+} of PinkyCaMP0.9a (**a**) and PinkyCaMP0.9b (**d**). (**b**,**e**) Absorbance spectra in the presence (39 µM) and absence of Ca^{2+} of PinkyCaMP0.9a (**b**) and PinkyCaMP0.9b (**e**). (**c**,**f**) Ca^{2+} titration curve (n = 3 replicates; mean ± s.d.) of

PinkyCaMP0.9a (**c**) and PinkyCaMP0.9b (**f**). To summarize, in the presence of Ca^{2+}, PinkyCaMP0.9a has absorbance and emission peaks at 569 and 596 nm, respectively. It exhibits a Ca^{2+}-dependent $\Delta F/F$ of 3.4 and has an apparent K_d for Ca^{2+} of 149 nM. The fluorescence response is primarily due to an increase in the quantum yield (0.16 to 0.44). The extinction coefficient remains fairly constant, only increasing from 89,000 $M^{-1}cm^{-1}$ to 98,000 $M^{-1}cm^{-1}$ upon binding to Ca^{2+} . Relative to PinkyCaMP0.9a, PinkyCaMP0.9b exhibited slightly red-shifted absorbance and emission peaks at 570 and 606 nm, respectively. It has a Δ *F/F of* 21.9 and an apparent K_d of 202 nM. As with PinkyCaMP0.9a, the fluorescence response is entirely attributed to a large quantum yield change from 0.02 to 0.43, with the extinction coefficient actually decreasing from 85,000 $M^{-1}cm^{-1}$ to 57,000 $M^{-1}cm^{-1}$, upon binding to Ca^{2+} (**Table 1**). (**g**) Brightness of PinkyCaMP0.9b (258.3±251.8a.u., n=154) and PinkyCaMP (456.7±456.4a.u., n=80) in HEK cells. Mean ± s.d.. Mann-Whitney U test, *p=0.0355. (**h-k**) Averaged photostability curves curves of 1µM of purified protein with 10mM EDTA or 10mM Ca^{2+}. Raw traces (**h-i**) of photobleaching with 57mW/mm^2 intensity and the same measurements normalized against extinction coefficient of each respective state (**j-k**). Mean ± s.e.m., n=3 replicates for each sensor and condition.

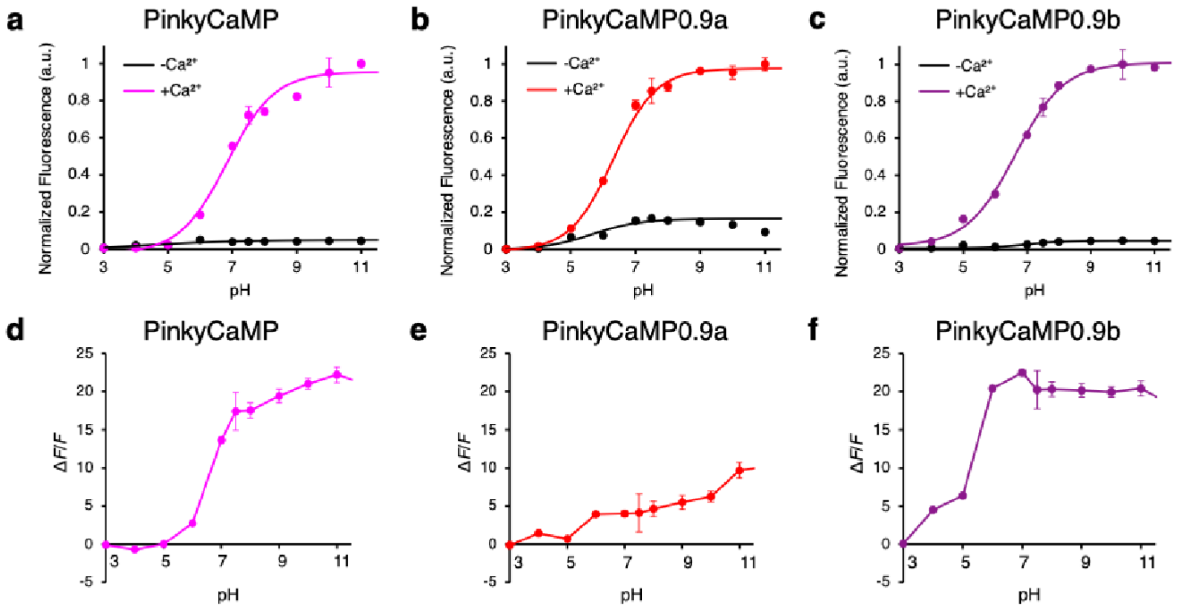

Supplementary Figure 3: pH dependence of PinkyCaMP variants. (**a**,**b**,**c**) pH titration curves in the presence (39 µM) and absence of Ca^{2+} (n = 3 replicates; mean ± s.d.) for PinkyCaMP (= PinkyCaMP0.9c) (**a**), PinkyCaMP0.9a (**b**), and PinkyCaMP0.9b (**c**). (**d**,**e**,**f**) pH-dependence of the Ca^{2+}-dependent Δ*F*/*F* for PinkyCaMP (**d**), PinkyCaMP0.9a (**e**) and PinkyCaMP0.9b (**f**), calculated by dividing the fluorescence intensity in the presence of Ca^{2+} by the fluorescence intensity in the absence of Ca^{2+}, at a particular pH value. Lines connecting the data points are simply to guide the eye.

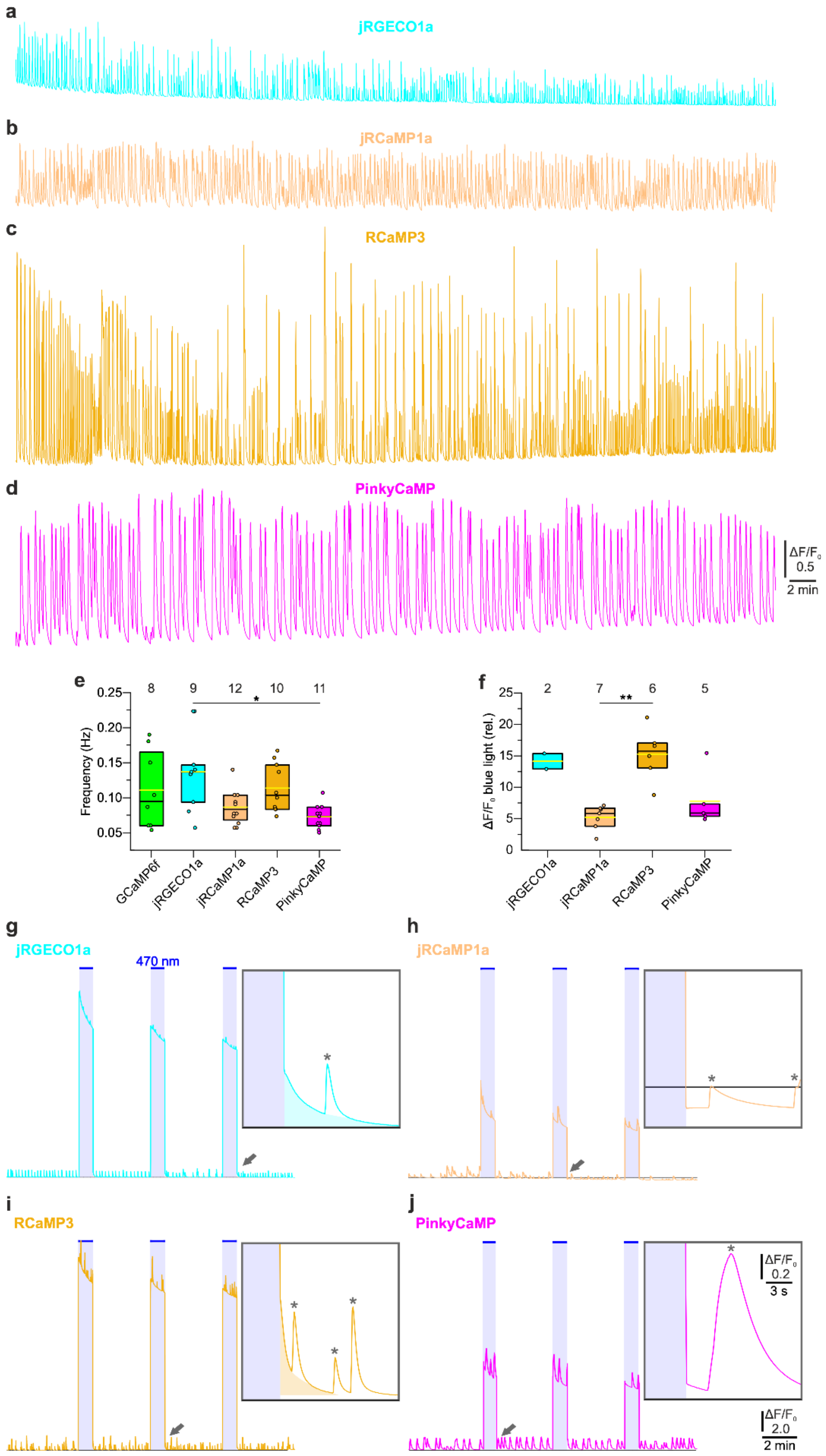
a
jRGECO1a
b
jRCaMP1a
c
RCaMP3
d
PinkyCaMP
ΔF/F₀
0.5
2 min
e
Frequency (Hz)
0.25
0.20
0.15
0.10
0.05
8
9
12
10
11
*
GCaMP6f
jRGECO1a
jRCaMP1a
RCaMP3
PinkyCaMP
f
ΔF/F₀ blue light (rel.)
25
20
15
10
5
0
2
7
6
5
**
jRGECO1a
jRCaMP1a
RCaMP3
PinkyCaMP
g
jRGECO1a
470 nm
*
h
jRCaMP1a
*
*
i
RCaMP3
*
*
*
j
PinkyCaMP
*
ΔF/F₀
0.2
3 s
ΔF/F₀
2.0
2 min

Supplementary Figure 4: Characterization of PinkyCaMP and other GECIs in organotypic slice cultures. (a-d) Example traces (60 min, $\Delta F/F_0$) of jRGECO1a, jRCaMP1a, RCaMP3 and PinkyCaMP showing spontaneous synchronous network activity at DIV 13-21. **(e)** Frequencies of synchronous activity for the slices represented in **Fig. 3** (for details see **Methods**). **(f-j)** Blue-light photoswitching experiments in organotypic slice cultures. **(f)** Fluorescence signal (emission 630 ± 38 nm) during high-intensity blue-light excitation. Blue light excites fluorescence more efficiently in jRGECO1a and RCaMP3 than in jRCaMP1a and PinkyCaMP. **(g-j)** Examples of continuous imaging with green-light excitation (554 nm) and three additional high-intensity blue-light stimulations (470 nm, 50 s each as indicated, for details see **Methods)**. The inserts show the first 14 s after the blue light is turned off. jRGECO1a and RCaMP3 show a decaying signal (colored background), which indicates recovery after photoswitching. These signals could be mistaken as Ca^{2+} elevations as they have similar amplitude as synchronous events (labeled with *). jRCaMP1a shows a slightly bleached signal, whereas PinkyCaMP shows a stable signal after the light is turned off (synchronous events again labeled with *). For quantification see **Fig. 3g**. Black boxes indicate 25-75% percentiles and medians, the yellow lines means. The numbers give the numbers of analyzed slices. Statistical differences were obtained by Dunn`s multiple comparisons after Kruskal-Wallis ANOVAs ($p < 0.05$) and are indicated with *$p \leq 0.05$ and **$p \leq 0.01$.

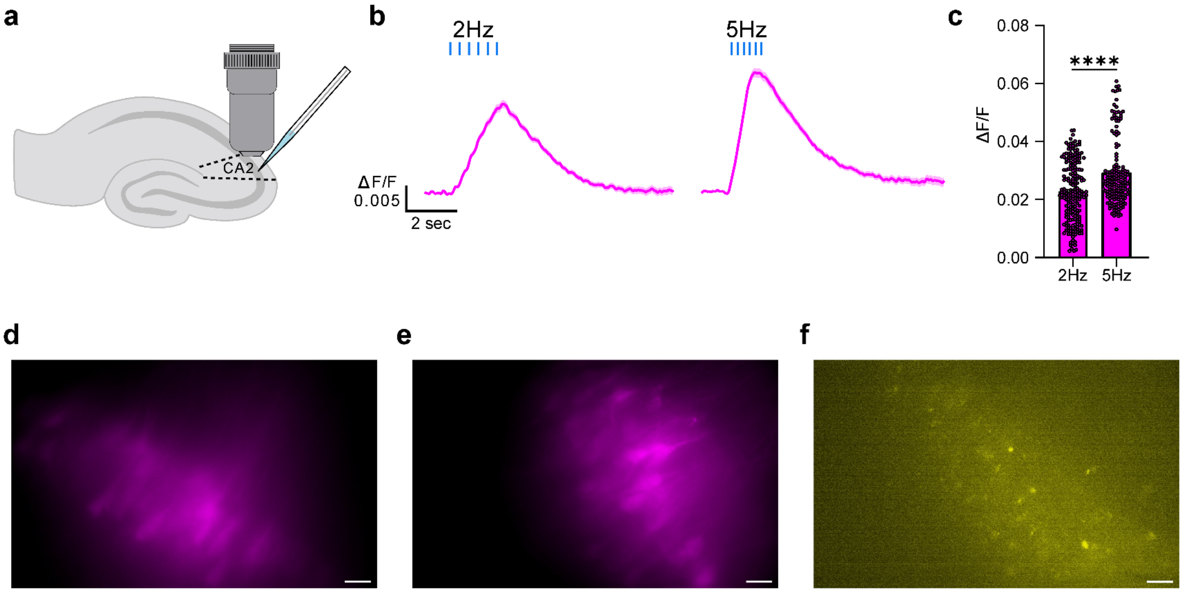

Supplementary Figure 5: Comparison of PinkyCaMP to other red GECIs in brain slices. (a) Schematic drawing of the stimulation and recording experimental design (b) Average Δ*F*/*F* traces of 6 stimulation pulses at 2Hz (left), n=204 cells, and 5Hz (right), n=167 cells, stimulation. (c) Maximal Δ*F*/*F* values for the traces in S7(b) (0.0228 ± 0.0007 and 0.0290 ± 0.0009 for 2Hz and 5Hz, respectively). Mann-Whitney U test, two-sided, ****p ≤ 0.0001. (d) Widefield image of PinkyCaMP in CA2 with 0.23 mW/mm² light intensity, (e) PinkyCaMP in CA2 with 11.83 mW/mm² light intensity, and (f) RCaMP3 in CA2 with 11.83 mW/mm² light intensity, scale bars 20 μm.
