# Supplementary material for: PinkyCaMP: an mScarlet-based calcium sensor with enhanced brightness, photostability and multiplexing capabilities
Source: Nat Methods. 2026 Apr 24;23(5):998–1010. doi: 10.1038/s41592-026-03065-2 (PMC13167472; doi:10.1038/s41592-026-03065-2)
Supplement: Supplementary file 2 — Reporting Summary [file 41592_2026_3065_MOESM2_ESM.pdf]

nature portfolio

Corresponding author(s): Olivia Masseck

Last updated by author(s): 17.12.2024

Reporting Summary

Nature Portfolio wishes to improve the reproducibility of the work that we publish. This form provides structure for consistency and transparency in reporting. For further information on Nature Portfolio policies, see our Editorial Policies and the Editorial Policy Checklist.

Statistics

For all statistical analyses, confirm that the following items are present in the figure legend, table legend, main text, or Methods section.

n/a	Confirmed	
☐	☒	The exact sample size (*n*) for each experimental group/condition, given as a discrete number and unit of measurement
☐	☒	A statement on whether measurements were taken from distinct samples or whether the same sample was measured repeatedly
☐	☒	The statistical test(s) used AND whether they are one- or two-sided<br>*Only common tests should be described solely by name; describe more complex techniques in the Methods section.*
☐	☒	A description of all covariates tested
☐	☒	A description of any assumptions or corrections, such as tests of normality and adjustment for multiple comparisons
☐	☒	A full description of the statistical parameters including central tendency (e.g. means) or other basic estimates (e.g. regression coefficient) AND variation (e.g. standard deviation) or associated estimates of uncertainty (e.g. confidence intervals)
☐	☒	For null hypothesis testing, the test statistic (e.g. *F*, *t*, *r*) with confidence intervals, effect sizes, degrees of freedom and *P* value noted<br>*Give P values as exact values whenever suitable.*
☒	☐	For Bayesian analysis, information on the choice of priors and Markov chain Monte Carlo settings
☒	☐	For hierarchical and complex designs, identification of the appropriate level for tests and full reporting of outcomes
☒	☐	Estimates of effect sizes (e.g. Cohen's *d*, Pearson's *r*), indicating how they were calculated

Our web collection on statistics for biologists contains articles on many of the points above.

Software and code

Policy information about availability of computer code

Data collection

For imaging in HEK cells and brain slices: LNscope from Luigs&Neumann equipped with a CMOS camera (Hamamatsu), Primary disscociated hippocampal neuronal culture: experiments were performed on an Olympus BX51 upright microscope equipped with a LUMPlanFL/IR ×40/0.80W objective.A Multiclamp 700B (Molecular Devices) and Digidata 1550B digitizer (both Molecular Devices) were used to control and acquire electrophysiological recordings as well as light engine LEDs, bipolar field stimulation and camera exposure timing. Field stimuli were applied thorugh a Warner Instrument SIU-102 Stimulus isolator.For action spectra recordings, light from the Lumencor SpectraX23 light engine was filtered with narrow bandpass filters mounted on a FW212C filter wheel (Thorlabs) and delivered to the sample plane using a FM03R cold mirror (Thorlabs) in the epifluorescence beam path. The following filters were used (center wavelength ± 10nm, Edmund Optics catalog no.): 372 nm (12147), 400 nm (65071), 422 nm (34496), 450 nm (65079), 480 nm (65084), 505 nm (34505), 535 nm (65095), 568 nm (65099), 600 nm (65102), 632 nm (65105) and 660 nm (86086). Imaging was performed with a Hamamatsu ORCA-Fire digital CMOS camera (C16240-20UP) .
Organotypic slices cultures:Ca2+ Imaging experiments were in a custom recording chamber (1.5 ml) and superfused with Ringer’s solution at 1 ml/min at 24 °C using a peristaltic pump (Minipuls 3, Gilson) and an in-line heater, respectively. The chamber was placed under an upright microscope (Axioscope, Zeiss) fitted with a 10x/0.3 water immersion objective (W N-Achroplan, Zeiss). Epifluorescence excitation for all red fluorescent indicators was provided by a collimated 554 nm LED (MINTL5, Thorlabs) using a 560/40 nm excitation filter and a 585 nm dichroic mirror, while fluorescence was collected with a 630/75 nm emission filter (ET-TxRed filter set, Chroma).
: DMi8 Leica with EMCCD camera (Evolve 512 delta, Photometrics), Confocal Microscopy LSM880 from Zeiss, 2P Imaging in vivo:For acquisition a custom-made Thorlaps two-photon microscope connected with a titanium sapphire 80 MHz Cameleon Ultra II two-photon lase (Coherent, Inc.) and equipped with an 8 kHz galvo-resonant scanner (LSK.GR08/M, Thorlabs), a GaAsP PMT (Thorlabs) and a 16x water immersion objective (Nikon) was used.In vivo fiber photometry Ca2+ signals (PinkyCaMP) and serotonin dynamics (sDarken) were recorded using an RX10x LUX-I/O Processor and Synapse software (TDT). An integrated LED driver controlled three LEDs for excitation: 560 nm (Lx560, TDT) for PinkyCaMP and mCherry, 465 nm (Lx465, TDT) for sDarken and 0Mut-sDarken, and 405 nm (LX405, TDT) for the isosbestic control

signal. Each wavelength was set to a light intensity of 25-30 µW and modulated at unique frequencies—530 Hz for 560 nm, 330 Hz for 465 nm, and 210 Hz for 405 nm. The LEDs were connected to a 6-port Fluorescence Mini Cube (Doric Lenses), with output delivered via a fiber-optic patch cord (NA: 0.48, 600 µm; Thorlabs) through a rotary joint (RJ1 1x1, Thorlabs) to a subject cable secured to the implanted ceramic fiber-optic cannula using an interconnect (ADAL2, Thorlabs).
Fiber photometry: Fiber photometry recordings in the dorsal DG were performed using an iFMC6_IE(400-410)_E1(460-490)_F1(500-540)_E2(555-570)_F2(580-680)_S photometry system (Doric Lenses) controlled by the Doric Neuroscience Studio v6.1.2.0 software. A low-autofluorescence patch cord (400 µm, 0.57 N.A., Doric Lenses) was attached to the metallic ferrule on mouse's head

Data analysis

ImageJ (Schneider et al.2012), IgorPro (WaveMetrics), ProfFit 7.0 (QuantumSoft),GraphPadPrism 9.3.1.Selfwritten Data analysis for dual color fiberphotometry is available on Github, other selfwritten codes will be made available upon request.The Fiber Photometire analysis script is publicly available on GitHub (https://github.com/masseck/FibPho-PinkyCaMP.git).

For manuscripts utilizing custom algorithms or software that are central to the research but not yet described in published literature, software must be made available to editors and reviewers. We strongly encourage code deposition in a community repository (e.g. GitHub). See the Nature Portfolio guidelines for submitting code & software for further information.

Data

Policy information about availability of data

All manuscripts must include a data availability statement. This statement should provide the following information, where applicable:

- Accession codes, unique identifiers, or web links for publicly available datasets
- A description of any restrictions on data availability
- For clinical datasets or third party data, please ensure that the statement adheres to our policy

DNA sequences are available in the Supplementary Information. DNA plasmids used for viral production have been deposited both on the UZH Viral Vector Facility (https://vvf.ethz.ch/) and on AddGene (plasmid #: 232857-232861). Viral vectors can be obtained either from the UZH Viral Vector Facility or from the Masseck lab. Raw data can be obtained by emailing the corresponding author. Source data are provided with this paper.

Human research participants

Policy information about studies involving human research participants and Sex and Gender in Research.

Reporting on sex and gender

Use the terms sex (biological attribute) and gender (shaped by social and cultural circumstances) carefully in order to avoid confusing both terms. Indicate if findings apply to only one sex or gender; describe whether sex and gender were considered in study design whether sex and/or gender was determined based on self-reporting or assigned and methods used. Provide in the source data disaggregated sex and gender data where this information has been collected, and consent has been obtained for sharing of individual-level data; provide overall numbers in this Reporting Summary. Please state if this information has not been collected. Report sex- and gender-based analyses where performed, justify reasons for lack of sex- and gender-based analysis.

Population characteristics

Describe the covariate-relevant population characteristics of the human research participants (e.g. age, genotypic information, past and current diagnosis and treatment categories). If you filled out the behavioural & social sciences study design questions and have nothing to add here, write "See above."

Recruitment

Describe how participants were recruited. Outline any potential self-selection bias or other biases that may be present and how these are likely to impact results.

Ethics oversight

Identify the organization(s) that approved the study protocol.

Note that full information on the approval of the study protocol must also be provided in the manuscript.

Field-specific reporting

Please select the one below that is the best fit for your research. If you are not sure, read the appropriate sections before making your selection.

☒ Life sciences ☐ Behavioural & social sciences ☐ Ecological, evolutionary & environmental sciences

For a reference copy of the document with all sections, see nature.com/documents/nr-reporting-summary-flat.pdf

Life sciences study design

All studies must disclose on these points even when the disclosure is negative.

Sample size

Sample size was not predetermined. Sampel size is given for each experiment

Data exclusions

No data have been excluded from the analysis.

Replication	Each of the described experiments are replicated in several dishes, cultures, slices or animals.
Randomization	Mice were randomly assigned in experimental groups. Mice of both sexes were used.
Blinding	Investigators were not blinded to the data or experimental animals.

Reporting for specific materials, systems and methods

We require information from authors about some types of materials, experimental systems and methods used in many studies. Here, indicate whether each material, system or method listed is relevant to your study. If you are not sure if a list item applies to your research, read the appropriate section before selecting a response.

Materials & experimental systems

n/a	Involved in the study
☐	☒ Antibodies
☐	☒ Eukaryotic cell lines
☒	☐ Palaeontology and archaeology
☐	☒ Animals and other organisms
☒	☐ Clinical data
☒	☐ Dual use research of concern

Methods

n/a	Involved in the study
☒	☐ ChIP-seq
☒	☐ Flow cytometry
☒	☐ MRI-based neuroimaging

Antibodies

Antibodies used	Primary antibody against GFP and mCerulean (chicken a-GFP, 1:1000; catalog no. GFP-1010, Aves Labs) andC-Fos rat a-cFOS, 1:1000; catalog no. 226 017, Synaptic Systems).<br>Secondary antibodies: Alexa Fluor 488 donkey anti-chicken, 1:500; catalog no. 703-545-155, Jackson laboratories and Alexa Fluor 647 goat anti-rat, 1:500; catalog no. 31226, Invitrogen, conjugated in-house)
Validation	Validation done by the manufactorer.

Eukaryotic cell lines

Policy information about cell lines and Sex and Gender in Research

Cell line source(s)	HEK293T (DSMZ ACC-635), HEK293T (Sigma Aldrich 12022001)
Authentication	none of the cell lines were authentificated
Mycoplasma contamination	Cells were are tested for Mycoplasma contamination on a regular basis.
Commonly misidentified lines<br>(See ICLAC register)	n.a.

Animals and other research organisms

Policy information about studies involving animals; ARRIVE guidelines recommended for reporting animal research, and Sex and Gender in Research

Laboratory animals	C57BL/6J mice of both sexes and vGAT mice (B6J.129S6(FVB)-Slc32a1tm2(cre)Lowl/MwarJ
Wild animals	n.a.
Reporting on sex	Sex was not considered in the study design.
Field-collected samples	n.a.
Ethics oversight	Animal protocols were approved by local authorities.

Note that full information on the approval of the study protocol must also be provided in the manuscript.
